# Induction of Cyp2e1 contributes to asparaginase-induced hepatocyte sensitization to lipotoxicity

**DOI:** 10.1016/j.apsb.2024.11.002

**Published:** 2024-11-07

**Authors:** Yin Zhu, Yuyin Wang, Keito Hoshitsuki, Da Yang, Lauren Kokai, Xiaochao Ma, Wen Xie, Christian A. Fernandez

**Affiliations:** a Center for Pharmacogenetics and Department of Pharmaceutical Sciences, University of Pittsburgh, Pittsburgh, PA 15261, USA; b Department of Plastic Surgery, University of Pittsburgh and the McGowan Institute for Regenerative Medicine, Pittsburgh, PA 15261, USA

**Keywords:** Liver injury, Asparaginase, Lipolysis, Lipotoxicity, Oxidative stress, ATGL, Cyp2e1

## Abstract

One of the leading therapies for acute lymphoblastic leukemia (ALL) is the chemotherapeutic agent PEGylated *E. coli*-derived-l-asparaginase (PEG-ASNase). Due to the high risk of dose-limiting liver injury, characterized by clinically elevated levels of hepatic transaminases, PEG-ASNase therapy is generally avoided in adult patients. Our preclinical investigations have indicated that PEG-ASNase-induced liver injury is associated with the release of free fatty acids (FFAs) from white adipose tissue (WAT), suggesting potential lipotoxic effects. However, it remains uncertain whether PEG-ASNase directly induces hepatotoxicity or sensitizes hepatocytes to FFA-induced toxicity. Our results show that PEG-ASNase treatment results in hepatocyte apoptosis and lipid peroxidation. *Ex vivo* and *in vitro* studies in mouse and human WAT suggest that PEG-ASNase induces the expression of adipose triglyceride lipase (ATGL), activates the lipase, and stimulates adipose tissue lipolysis, suggesting that the FFAs from WAT may contribute to the observed liver injury. Moreover, treatment with PEG-ASNase sensitizes hepatocytes to FFA-induced lipotoxicity. Mechanistically, our RNA-sequencing (RNA-seq) analyses reveal that PEG-ASNase-induced sensitization to lipotoxicity is accompanied by the induction of Cyp2e1. We demonstrated that this sensitization effect is attenuated by both pharmacological and genetic inhibition of Cyp2e1. Our findings suggest that PEG-ASNase therapy induces WAT lipolysis and sensitizes hepatocytes to hepatic lipotoxicity in a Cyp2e1-dependent manner.

## Graphical abstract

PEGylated asparaginase (PEG-ASNase) induces white adipose tissue lipolysis *via* the activation of ATGL and sensitizes hepatocytes to lipotoxicity through the upregulation of Cyp2e1.

**Figure:**
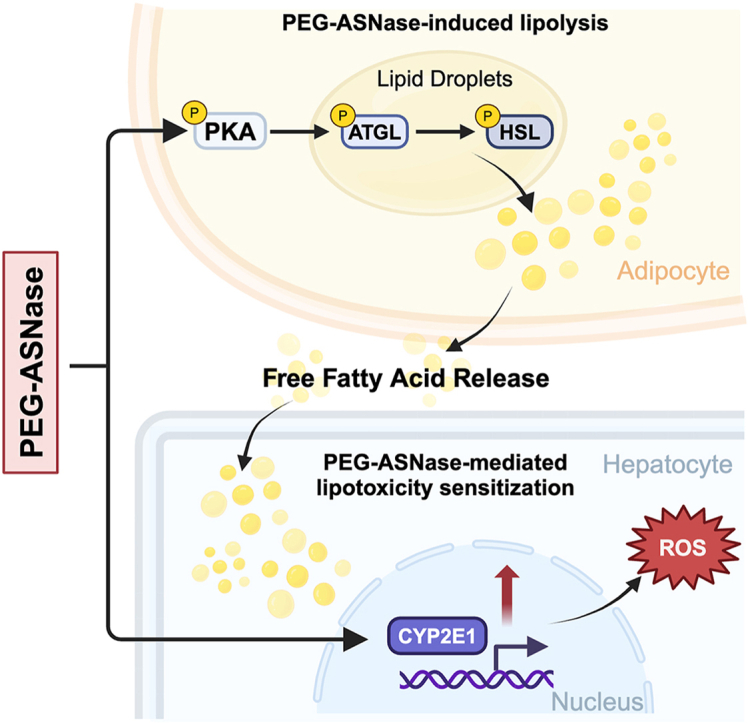

## 1 Introduction

Acute lymphoblastic leukemia (ALL) cells are unable to synthesize asparagine *de novo*, and therefore are susceptible to asparagine depletion by PEGylated *E. coli* asparaginase (PEG-ASNase)^1^. While PEG-ASNase is used as first-line therapy for pediatric ALL^2^, it is often excluded in adult ALL treatment protocols because it is associated with a high risk of drug-induced liver injury^3^. Clinical studies assessing the safety of ASNase demonstrated that adult ALL patients can develop elevations in serum biomarkers of liver injury, such as alanine aminotransferase (ALT) *via* an unknown mechanism^4^. Nevertheless, recent clinical data in adolescents and young adults demonstrated a nearly 2-fold higher survival rate for ALL patients treated with pediatric-inspired treatment regimens including ASNase^5^. Therefore, strategies that reduce the risk of liver injury induced by ASNase and ensure its safe use may positively impact patient survival rates.

While the mechanism of ASNase-induced liver injury is unknown, our clinical studies have identified genetic risk factors for the toxicity, such as the *PNPLA3* I148M variant^6^. Additionally, our preclinical research in mice demonstrated that the agent is not directly toxic to hepatocytes, but rather is associated with the mobilization of FFAs from the adipose tissue^7^. Both our genetic and preclinical studies suggest a link between ASNase-induced liver injury and mechanisms of hepatic steatosis, implying a potential pathway to liver damage through the development of fatty liver.

Based on our previous studies demonstrating increased adipocyte adipose triglyceride lipase (ATGL) expression after PEG-ASNase treatment^7^, we hypothesize that PEG-ASNase activates ATGL, leading to enhanced lipolysis in adipose tissue and the accumulation of FFAs in the liver. Furthermore, because hepatic FFAs are not typically toxic to hepatocytes, we posit that PEG-ASNase sensitizes hepatocytes to lipid-induced toxicity or lipotoxicity. Herein, we describe preclinical studies demonstrating that ATGL is activated in adipocytes following PEG-ASNase treatment. The PEG-ASNase-induced phenotype is consistent in both mouse and human adipose tissue samples. Furthermore, we demonstrate that PEG-ASNase leads to lipotoxicity in mice and present data supporting that it sensitizes hepatocytes to lipotoxicity through the induction of Cyp2e1.

## 2 Materials and methods

### 2.1 Mice

Eight-week-old male and female C57BL/6 mice were purchased from Jackson Laboratories for our studies. Upon arrival, mice were acclimated for a minimum of three days prior to all experiments. Mice were fed with *ad libitum* standard mouse chow diet and water. All mice were housed in a specific pathogen-free animal facility under the supervision of the Division of Laboratory Animal Resources at the University of Pittsburgh with 12 h light and dark cycles. All experimental protocols were reviewed and approved by the University of Pittsburgh Institutional Animal Care and Use Committee and in compliance with the National Institutes of Health guide for the care and use of laboratory animals.

### 2.2 PEG-ASNase administration

Mice received a single dose of 1500 IU/kg PEG-ASNase (Oncaspar®) or PBS vehicle control intraperitoneally (IP) and were euthanized 3 days after administration similar to our previous study^7^. Blood, liver, and white adipose tissue (WAT) were collected after 6 h of fasting. Data were generated using 10 mice per group (5 males and 5 females).

### 2.3 Measurement of plasma biomarkers of liver injury and non-esterified fatty acid (NEFA)

Plasma liver injury biomarkers, including alanine aminotransferase (ALT), aspartate aminotransferase (AST), and NEFA, were measured using the Randox Imola clinical chemistry analyzer (Randox, UK), following the manufacturer's instructions.

### 2.4 Quantification of hepatic triglyceride concentrations

Hepatic triglyceride concentrations were measured as previously described^8^. Briefly, the liver tissue was weighed and homogenized in a polystyrene tube containing 1 mL of 5% Nonidet-P40 (United States Biological Corporation, 9036-19-5). The tissue homogenate was heated to 95 °C for 5 min and cooled for 2 cycles. Then homogenate was centrifuged for 5 min at 13,000 rpm (Sorvall Legend Micro 17R Centrifuge, Thermofisher, MA, USA). The supernatant was collected, and the concentration of triglycerides was measured using the Stanbio triglyceride assay kit (Stanbio Laboratory, 2100-225). Hepatic triglyceride concentrations are represented as mg/dL normalized to sample tissue weight.

### 2.5 Real-time quantitative PCR and Western blot analysis

Total RNA from tissues and/or cells was extracted with TRIzol reagent (Invitrogen, 16096040). Complementary DNA was synthesized, and SYBR Green-based real-time PCR was performed with Applied Biosystems QuantStudio 7 Pro Dx Real-Time PCR System. Raw data were normalized to the control *β*-actin (Actb) by the ΔΔCt method. PCR primer sequences are listed in Supporting Information Table S1. For Western blot protein analysis, 20 μg of protein extracts were separated by sodium dodecyl sulfate–polyacrylamide gel electrophoresis gels and transferred onto a polyvinylidene difluoride membrane. Primary antibodies are listed in Supporting Information Table S2. Quantification of protein bands was performed using ImageJ software.

### 2.6 Immunostaining and TUNEL analysis

Paraffin sections were deparaffinized, rehydrated, and stained using an anti-4-hydroxynonenal antibody (4-HNE, Thermofisher Scientific, BS-6313R) overnight at 4 °C. The VECTASTAIN ABC Kit and DAB Peroxidase Substrate Kit (Vector Laboratories, PK-4010 and SK-4105, respectively) were used to visualize the staining. Hematoxylin was used as a nuclear counterstain. The positive area percentage in each tissue section was assessed through the quantification of positive areas using ImageJ Software. For the TUNEL analysis, the sections were stained using a TUNEL Kit (Sigma–Aldrich, S7100) following the manufacturer's instructions.

### 2.7 Histological analysis

Murine liver tissues were fixed for 24 h in 10% formalin. After paraffin embedding, 4 μm tissue sections were sectioned using a microtome. The sections were deparaffinized and rehydrated for histological analysis using Hematoxylin and Eosin (H&E). To visualize lipid droplets, livers were embedded in an optimum cutting temperature compound right after sacrifice. 8 μm tissue sections were cut in a freezing microtome and were subjected to Oil red O (ORO) staining.

### 2.8 Primary murine hepatocyte isolation

Primary murine hepatocytes were isolated as previously described^9^. Briefly, 5 mice (with at least 2 males and 2 females) were anesthetized, and the inferior vena cava was cannulated with a 22G catheter and fixed with silk thread. Perfusion was performed using Solution A (Hank's Balanced Salt Solution (HBSS) without Ca^2+^/Mg^2+^, 0.2 mol/L EGTA, 1 mol/L HEPES buffer) at a flow rate of 5 mL/min for 10 min, followed by Solution B (Leibovitz's L-15 Medium, 0.5 mol/L CaCl_2_, 1 mol/L HEPES buffer, 2.5 mg/mouse Liberase^TM^ (Sigma, 5401127001) at a flow rate of 4 mL/min for 10 min. After perfusion, the liver was transferred to 10 mL ice-cold William E media and minced with forceps. The digested liver was filtered through a 100 μm cell strainer. Cells were centrifuged and seeded on collagenase-coated wells overnight before treatments. Media were changed to HepatoZYME-SFM media the following day.

For studies assessing whether PEG-ASNase sensitizes hepatocytes to FFA-induced toxicity, hepatocytes were treated with PEG-ASNase (1 IU/mL), palmitic acid (0.5 mmol/L), or both for 48 h. We further verified PEG-ASNase-induced sensitization to lipotoxicity by concomitant treatment with PEG-ASNase and 4-HNE (Cayman Chemical, 32100, 25 μmol/L^10^). To investigate if pharmacological inhibition of Cyp2e1 attenuates PEG-ASNase-induced lipotoxicity sensitization, a subset of hepatocytes was treated with PEG-ASNase and palmitic acid along with diallyl sulfide (Cayman Chemical, 20894, 400–500 μmol/L), similar to previous studies^11^^,^^12^. All data generated using primary murine hepatocytes are based on biological replicates and are represented as means ± standard deviation.

### 2.9 Cell culture and 3T3-L1 adipocyte differentiation

HepG2, a human hepatocellular carcinoma cell line, was grown in Eagle's minimum essential medium supplemented with 10% fetal bovine serum, penicillin (100 U/mL), and streptomycin (100 μg/mL) at 37 °C in humidified air with 5% CO_2_. Cells were treated with PEG-ASNase (1 IU/mL) and/or palmitic acid (0.5 mmol/L) for 48 h before subsequent analyses. To induce differentiation of 3T3-L1 mouse embryonic fibroblast cells into adipocytes, cells were plated in a 6-well plate and treated with a combination of 0.5 mmol/L isobutyl methylxanthine, 1 μmol/L dexamethasone, and 10 μg/mL insulin at 70% confluency. After three days, the induction media was changed to 1% insulin DMEM with 10% fetal bovine serum^7^. Differentiated adipocytes were treated with 1 IU/mL PEG-ASNase. After 72 h of treatment, conditioned media and cellular protein were harvested. HepG2 cells were treated with the conditioned media of 3T3-L1 cells and PEG-ASNase (1 IU/mL) for 48 h before subsequent analyses.

### 2.10 siRNA-mediated knockdown of Cyp2e1 in primary murine hepatocytes and HepG2 cells

Primary murine hepatocytes were seeded at densities of 1.0 × 10^4^ cells/mL in 96-well plates and 3.0 × 10^5^ cells/mL in 6-well plates (*n* = 3) and incubated overnight before transfection. Knockdown of Cyp2e1 expression was performed using siRNA sequences targeting the mouse *Cyp2e1* gene (siRNA ID: 161877, Ambion, Austin, TX, USA). The following pairs of 21-bp oligonucleotide sequences were annealed and reconstituted in RNase-free water: Forward: 5′-GGUUUUCCCUAAGUAUCCU-3′ and Reverse: 5′-AGGAUACUUAGGGAAAACC-3′. A pre-designed negative control siRNA (Invitrogen™, AM4611) was also used to account for off-target effects. Cells were transfected with 10 nmol/L of siRNA-Cyp2e1 using the TransIT-TKO Transfection Reagent (Mirus Bio, MIR 2154) for 48 h before subsequent analyses. A similar method was performed with HepG2 cells seeded at 1.0 × 10^4^ cells/mL in 96-well plates and 1.0 × 10^6^ cells/mL in 6-well plates (*n* = 3). HepG2 cells were transfected with specific human siRNA for CYP2E1 (Santa Cruz Biotechnology, sc-105257) and control siRNA (Santa Cruz Biotechnology, sc-37007) using Lipofectamine RNAiMax (Invitrogen™, 13-778-030).

### 2.11 Human adipose tissue explant study

Human adipose tissue was obtained from non-diabetic, non-smoker patients undergoing elective plastic surgery procedures at the University of Pittsburgh Medical Center (UPMC) Adipose Stem Cell Center, following similar methods described previously^13^. Detailed patient demographics are listed in Supporting Information Table S3. Informed written consent was obtained, and the experimental procedures were conducted in full compliance with the relevant Code of Ethics. Institutional Review Board approval was granted on November 1st, 2023. Approximately 0.5 mL samples were distributed into 1.9 cm^2^ wells incubated with DMEM/F12 supplemented with 5% fetal bovine serum, penicillin (100 U/mL), and streptomycin (100 μg/mL) at 37 °C in humidified air with 5% CO_2_. Human adipose tissue samples were treated with either 1 IU/mL PEG-ASNase or vehicle media. Conditioned media and fat particles were collected at 24, 48, and 72 h and subjected to assays for NEFA concentration, protein analysis, and RNA expression, respectively.

### 2.12 Intracellular reactive oxidative stress (ROS) detection

Intracellular ROS levels were detected using a DCFDA Cell-based Assay Kit (Cayman, 601520). Briefly, treated primary hepatocytes were stained with ROS staining buffer, with negative and positive controls assigned to *N*-acetyl cysteine assay reagent and pyocyanin, respectively. Following the staining process, cells were incubated at 37 °C for 1.5 h under light protection. Fluorescence intensity was measured using an excitation wavelength of 485 nm and an emission wavelength of 528 nm.

### 2.13 MTT cell viability assay

A total of 1.0 × 10^4^–2.0 × 10^4^ cells per well were seeded in 96-well culture plates in triplicate (*n* = 3) for a 48 h exposure to chemical treatments with 100 μL culture medium. Following this incubation period, 10 μL of a 12 mmol/L solution of Thiazolyl Blue Tetrazolium Bromide (Biosynth, T-3450) was added to each well. The plates were then incubated at 37 °C for 2–4 h. Once purple precipitation was clearly visible, 100 μL of isopropanol with 0.3% HCl was added to dissolve the formazan. The absorbance was measured at 570 nm.

### 2.14 RNA-sequencing data analysis

Treated primary murine hepatocytes were washed with cold-PBS twice before total RNA isolation using TRIzol reagent (Invitrogen, 16096040) and subsequently purified with the RNA Clean & Concentrator Kit (Zymo Research, R1017), resulting in a yield of up to 100 μg of total RNA. RNA-sequencing and data analysis were performed by Novogene Corporation Inc. Briefly, the sequence libraries were generated using the NEBNext® Ultra™ RNA Library Prep kit (Illumina®, NEB, USA), and cDNA purification was carried out using the AMPure Xp system (Beckman Coulter, Brea, CA, USA). The quality of the cDNA library was assessed with an Agilent Bioanalyzer 2100 system. Clean paired-end reads were aligned to the mouse (Mus musculus) reference genome (GRCm38/mm10) using HISAT2 software^14^. Gene expression levels were estimated based on transcript abundance mapped to the genome or exon. Log_2_ fold changes of differentially expressed genes were determined using DESeq2 package^15^, with genes exhibiting *P* < 0.05 considered significantly expressed. Gene ontology enrichment analysis, including the Kyoto Encyclopedia of Genes and Genomes (KEGG) pathway, was conducted using the ClusterProfiler R package.

### 2.15 Statistical analysis

The results are presented as mean ± standard deviation (SD). Statistical significance was assessed through Student's *t*-tests, estimated using GraphPad Prism 10.1 (GraphPad Software, CA, USA). ∗*P* < 0.05; ∗∗*P* < 0.01; ∗∗∗*P* < 0.001; ∗∗∗∗*P* < 1 × 10^−4^. A significance level of *P* < 0.05 was considered indicative of statistical significance.

## 3 Results

### 3.1 Mice receiving PEG-ASNase develop lipotoxicity

**Figure 1:**
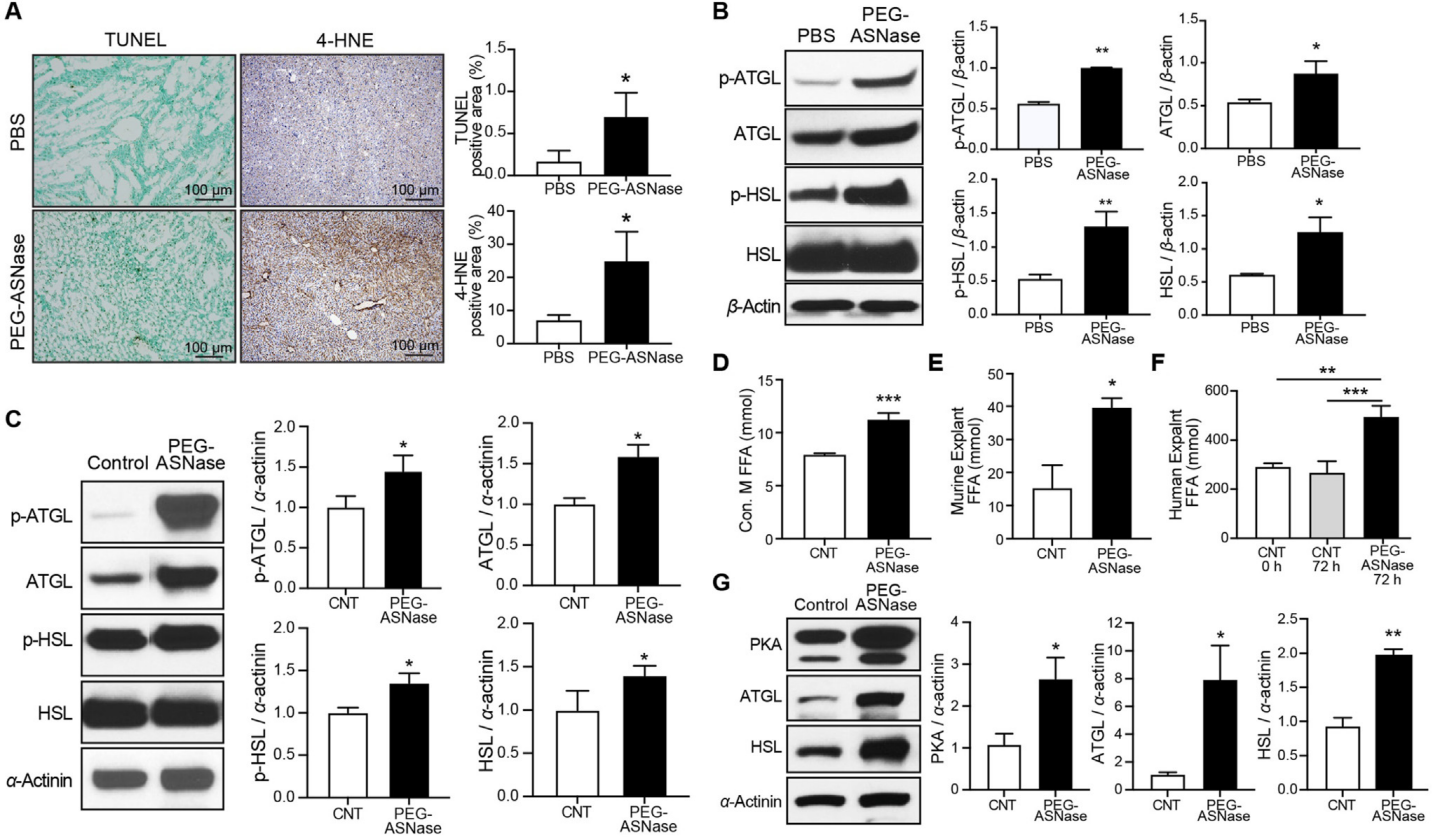
PEG-ASNase treatment led to hepatic lipotoxicity, increased phosphorylated ATGL in white adipose tissue (WAT), and induced FFA mobilization in both murine and human samples. (A) PEG-ASNase treatment led to a significant increase in hepatic TUNEL and 4-HNE staining relative to PBS controls. (B) The protein levels of phosphorylated-ATGL (p-ATGL), ATGL, phosphorylated-HSL (p-HSL), and HSL in WAT were elevated in mice treated with PEG-ASNase compared to PBS. (C) Similar results were shown in differentiated 3T3-L1 adipocytes treated for 72 h with 1 IU/mL of PEG-ASNase. (D) Conditioned media (Con. M) from differentiated 3T3-L1 adipocytes treated with PEG-ASNase showed higher FFA concentrations relative to controls. (E) PEG-ASNase treatment of *ex vivo* mouse WAT explants increased the FFA concentration in the conditioned media compared to control media at 24 h. (F) Human WAT explants treated with 1 IU/mL PEG-ASNase demonstrated a similar release of FFA compared to vehicle control. (G) In human WAT, the protein levels of PKA, ATGL, and HSL were elevated after PEG-ASNase treatment compared to the vehicle. ∗, ∗∗, ∗∗∗ indicate *P* < 0.05, 0.01, and 0.001, respectively.

Our previous study investigating the mechanism of PEG-asparaginase-induced liver injury demonstrated that the antileukemic agent leads to the development of fatty liver. The liver injury observed was associated with the mobilization of FFAs from adipose tissue and the upregulation of ATGL, which is involved in triglyceride hydrolysis^7^. Based on our previous observations, we hypothesized that the liver injury in mice resulted from increased sensitivity to lipid-mediated toxicity, or lipotoxicity. To assess our hypothesis, C57BL/6J mice were administered a single IP dose of 1500 IU/kg PEG-ASNase. Mice exhibited a loss in body and liver weight, elevated ALT and AST levels, and developed hepatic steatosis, along with a loss of WAT mass and increased NEFA plasma levels, similar to findings from our previous study (Supporting Information Fig. S1A–S1H, *P* < 0.01)^7^. Given the potential toxicity of FFA to hepatocytes, we next investigated whether PEG-ASNase treatment induced hepatic lipid peroxidation in our mouse model. Lipid peroxidation, or oxidative degradation of lipids, can lead to cell membrane disruption and lipid-mediated hepatocyte apoptosis^16^. To assess whether the mobilization of FFAs associated with PEG-ASNase treatment leads to hepatic lipid peroxidation in mice, we measured the hepatic levels of 4-HNE and determined hepatocyte apoptosis using the TUNEL assay. Our results show that PEG-ASNase increased 4-HNE and TUNEL staining relative to the vehicle control mice (Fig. 1A, *P* < 0.05), indicating that PEG-ASNase may lead to hepatic lipotoxicity and suggesting that the antileukemic agent may sensitize hepatocytes to lipid-mediated apoptosis.

### 3.2 PEG-ASNase increases p-ATGL in WAT and induces FFA mobilization in both mouse and human samples

Our previous studies have shown that PEG-ASNase induces ATGL^7^, which is consistent with our current observations showing that PEG-ASNase leads to a loss of WAT mass and an increase in plasma FFA levels. Based on our results, we posited that PEG-ASNase activates lipolytic lipases, including ATGL and hormone sensitive lipase (HSL) *via* a mechanism that has yet to be determined. Given that both ATGL and HSL are activated through phosphorylation^17^^,^^18^, we evaluated their activation following PEG-ASNase treatment by Western blot. Consistent with our current and previous studies, our results show increased levels of p-ATGL, ATGL, p-HSL, and HSL in PEG-ASNase-treated mice, compared to mice treated with PBS (Fig. 1B, *P* < 0.05), supporting the activation and induction of ATGL by PEG-ASNase (p-ATGL/ATGL ratio = 1.15; *P* = 0.07). A similar induction and phosphorylation of ATGL was demonstrated in differentiated 3T3-L1 adipocytes treated with PEG-ASNase (Fig. 1C, p-ATGL/ATGL ratio = 1.37; *P* < 0.05). Supporting that the activation of ATGL induces adipocyte FFA mobilization or lipolysis, we show that PEG-ASNase treatment leads to elevated FFA levels in the conditioned media from both differentiated 3T3-L1 adipocytes and *ex vivo* WAT explants from mice (Fig. 1D and E, *P* < 0.05). To investigate whether PEG-ASNase treatment can induce ATGL and HSL and promote adipocyte lipolysis in humans, we treated adipose tissue fat pads from non-diabetic human donors with clinically relevant concentrations of PEG-ASNase (1 IU/mL) or vehicle^19^, ^20^, ^21^. We show that PEG-ASNase increases FFAs in the conditioned media of human adipose tissue samples (Fig. 1F, *P* < 0.01) and upregulates the expression of protein kinase A (PKA), ATGL, and HSL (Fig. 1G, *P* < 0.05). These findings support that PEG-ASNase activates lipolytic lipases through a similar mechanism in both mice and humans.

### 3.3 PEG-ASNase sensitizes hepatocytes to fatty acid-induced toxicity

**Figure 2:**
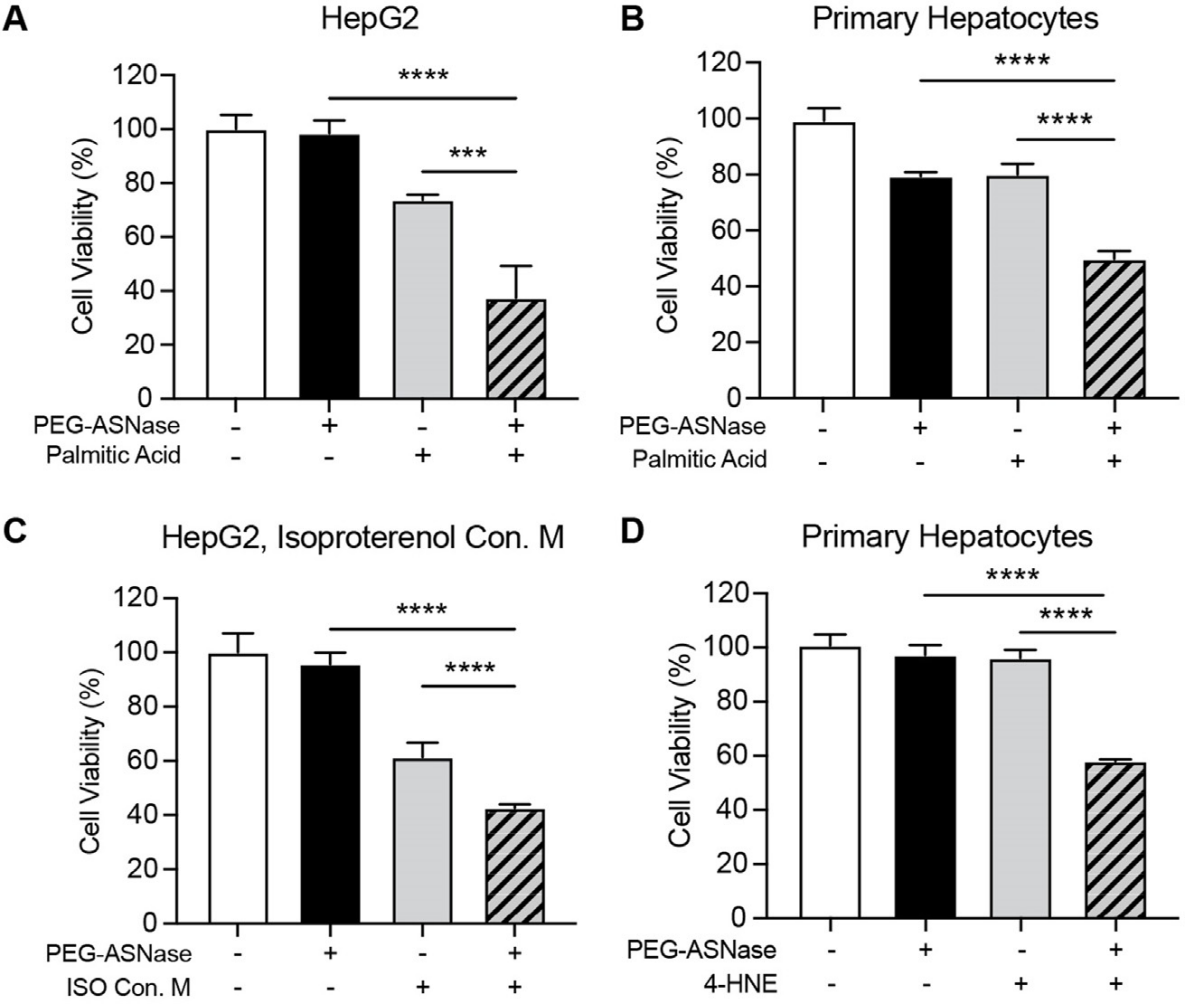
PEG-ASNase sensitizes hepatocytes to fatty acid-induced toxicity. (A) PEG-ASNase (1 IU/mL) sensitized HepG2 cells to palmitic acid (0.5 mmol/L)-induced lipotoxicity. (B) Primary murine hepatocytes treated with PEG-ASNase and palmitic acid (0.5 mmol/L) were also sensitized to lipotoxicity. (C) PEG-ASNase sensitized HepG2 cells to FFAs from the cultured conditioned media of isoproterenol-stimulated 3T3-L1 adipocytes (ISO Con. M). (D) Primary murine hepatocytes treated with PEG-ASNase are sensitized to 4-HNE-mediated toxicity (25 μmol/L) relative to single-agent treatments. ∗∗∗, ∗∗∗∗ indicate *P* < 0.001 and 1 × 10^−4^.

Based on our *in vivo* and *in vitro* studies suggesting that PEG-ASNase activates ATGL in WAT, induces adipocyte lipolysis, and leads to hepatic steatosis and hepatocyte apoptosis, we hypothesized that PEG-ASNase sensitizes hepatocytes to FFA-induced toxicity. To test this hypothesis, we treated primary hepatocytes and HepG2 cells with PEG-ASNase (1 IU/mL) and/or palmitic acid (PA, 0.5 mmol/L), which served as a physiologically relevant source of FFA to stimulate lipotoxicity^22^, for 48 h and assessed cell viability. We showed that PEG-ASNase or PA has little to no effect on cell viability, whereas the combination treatment of PEG-ASNase and PA leads to a decrease in cell viability relative to single-agent treatment (Fig. 2A and B, *P* < 1 × 10^−4^). To investigate whether FFA released from adipocytes following PEG-ASNase-induced lipolysis can sensitize hepatocytes to lipotoxicity, we induced lipolysis in 3T3-L1 adipocytes using isoproterenol. Subsequently, we collected conditioned media containing mobilized FFAs and treated HepG2 cells with this media, both with and without PEG-ASNase. Similar to our studies using PA, PEG-ASNase had no effect on HepG2 cell viability (*P* = 0.9839). In contrast, treating HepG2 cells with PEG-ASNase in combination with the conditioned media of lipolysis-stimulated adipocytes led to a sharp drop in cell viability relative to controls (Fig. 2C, *P* < 1 × 10^−4^). Given that the mechanism of lipotoxicity involves ROS-mediated cell damage^23^, we next assessed whether PEG-ASNase exacerbates toxicity directly mediated by 4-HNE, a highly reactive aldehyde generated as a byproduct of lipid peroxidation. Similar to the effects of PA or conditioned media from stimulated 3T3-L1 adipocytes, PEG-ASNase significantly sensitized primary hepatocytes to 4-HNE-mediated toxicity compared to the single-agent treatment controls (Fig. 2D, *P* < 1 × 10^−4^). Altogether, our findings support that PEG-ASNase sensitizes hepatocytes to lipotoxicity by potentiating the effects of lipid peroxidation byproducts on hepatocytes.

### 3.4 Upregulation of Cyp2e1 is associated with PEG-ASNase-induced lipotoxicity sensitization

**Figure 3:**
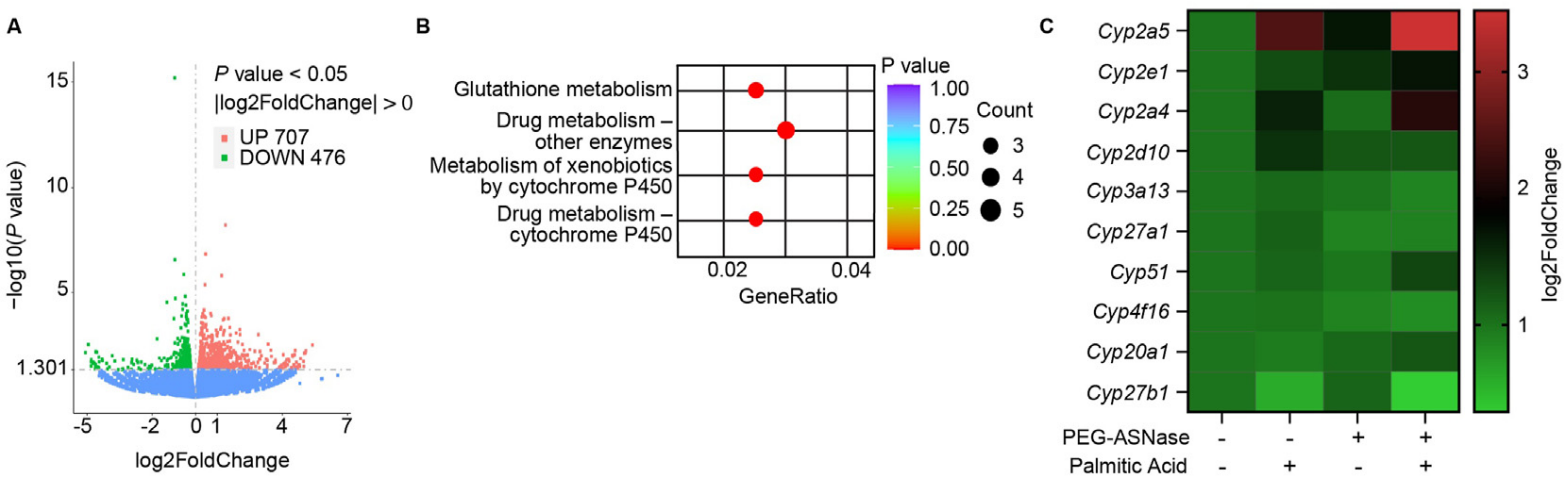
Upregulation of Cyp2e1 is associated with PEG-ASNase-induced lipotoxicity sensitization. (A) The volcano plot shows differential gene expression induced by the combination treatment of PEG-ASNase and palmitic acid compared to the palmitic acid alone group, with *P* values < 0.05. (B) Pathway analysis of differentially expressed genes showed that the top four significantly enriched KEGG pathways involve drug metabolism pathway. (C) The heatmap shows the expression changes in CYP enzyme genes following treatment with palmitic acid, PEG-ASNase, or the combination relative to vehicle control.

To elucidate the mechanisms underlying the PEG-ASNase-induced sensitization to lipotoxicity, we performed RNA-seq analysis on primary mouse hepatocytes treated with PA alone and in combination with PEG-ASNase. In our analysis, we identified 1183 dysregulated genes (*P* values < 0.05, 707 upregulated and 476 downregulated, Fig. 3A, Supporting Information Table S4). These genes were significantly enriched in key biological pathways including glutathione metabolism, drug metabolism mediated by cytochrome P450 (CYP), and the metabolism of xenobiotics by CYP (Fig. 3B, *P* < 0.05). Based on these results, we focused more closely on analyzing the differential expression of CYP enzymes between the treatment groups. We found that in hepatocytes treated with both PEG-ASNase and PA, 10 CYP enzymes were significantly dysregulated compared to those treated with PA alone (Fig. 3C,Table S4). Among these, Cyp2e1 expression was induced by PEG-ASNase treatment alone and further elevated in combination with PA, with only modest effects observed from PA treatment alone (Fig. 3C). Cyp2e1 is expressed mainly in the hepatocytes of the liver^24^, where it plays a crucial role in ethanol-induced oxidative stress^25^. It uses molecular oxygen to metabolize its substrates, leading to the production of ROS and lipid peroxidation^26^. Cyp2e1 metabolizes a variety of substrates, such as ethanol, acetaminophen, and saturated and polyunsaturated fatty acids^27^. The RNA-seq results suggest that the dysregulation of CYP enzymes by PEG-ASNase is associated with hepatocyte sensitization to FFA-induced toxicity and indicates the possible involvement of Cyp2e1 in the sensitization.

### 3.5 The induction of Cyp2e1 is associated with elevated ROS levels, while pharmacological and genetic inhibition of Cyp2e1 attenuates PEG-ASNase-induced lipotoxicity sensitization

**Figure 4:**
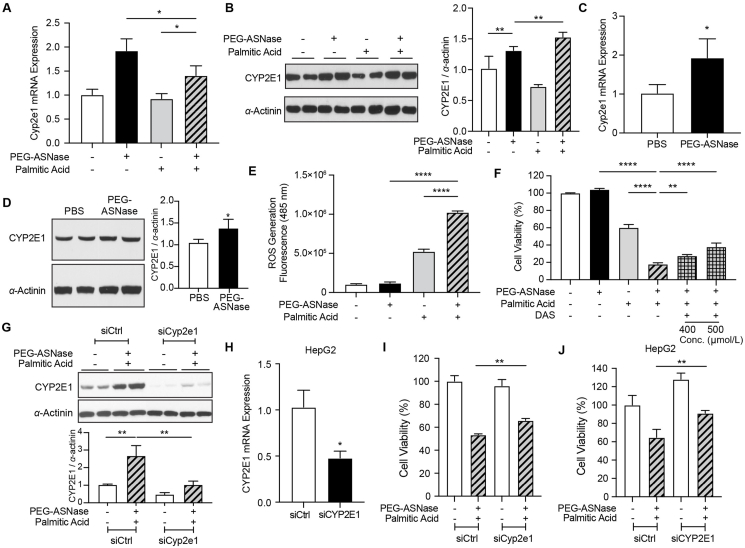
Pharmacological and genetic inhibition of Cyp2e1 mitigates hepatocyte sensitization to lipotoxicity by PEG-ASNase. (A, B) PEG-ASNase induces *Cyp2e1* mRNA and protein expression levels in primary murine hepatocytes. (C, D) Hepatic *Cyp2e1* mRNA and protein expression were upregulated in mice receiving PEG-ASNase. (E) Palmitic acid treatment increases ROS levels in primary murine hepatocytes, and this effect was further exacerbated by concurrent PEG-ASNase treatment. (F) The Cyp2e1 inhibitor, diallyl sulfide (DAS, 400–500 μmol/L) protects against PEG-ASNase-mediated lipotoxicity sensitization in a concentration-dependent manner. (G) Primary murine hepatocytes and (H) HepG2 cells treated with siRNA targeting Cyp2e1 exhibited decreased Cyp2e1 protein and mRNA levels, respectively, and (I, J) were protected against PEG-ASNase-induced lipotoxicity sensitization compared to siRNA control (siCtrl) groups. ∗, ∗∗, ∗∗∗∗ indicate *P* < 0.05, 0.01, and 1 × 10^−4^, respectively.

To validate our RNA-seq findings, we assessed the gene and protein expression of Cyp2e1 in primary mouse hepatocytes treated with PA alone and in combination with PEG-ASNase. We found that Cyp2e1 mRNA and protein levels are upregulated by PEG-ASNase treatment, irrespective of PA treatment (Fig. 4A and B, *P* < 0.05). Consistent with the results of our *in vitro* experiments, we observed a similar increase in *Cyp2e1* mRNA and protein expressions in the liver of mice treated with PEG-ASNase compared to PBS control (Fig. 4C and D, *P* < 0.05). Given the induction of Cyp2e1 by PEG-ASNase and its involvement in ROS generation, we hypothesized that PEG-ASNase treatment would exacerbate PA-induced ROS production in hepatocytes. Consistent with our hypothesis, our results indicated that PEG-ASNase potentiates the generation of ROS by PA (Fig. 4E, *P* < 1 × 10^−4^). To further substantiate the role of PEG-ASNase-induced Cyp2e1 in sensitizing hepatocytes to PA-induced lipotoxicity, we used a Cyp2e1 inhibitor, diallyl sulfide (DAS)^28^, to determine whether inhibiting the enzyme activity of Cyp2e1 protected against PEG-ASNase-induced lipotoxicity sensitization. Our results show that DAS (400 and 500 μmol/L) treatment attenuates hepatocyte sensitization to lipotoxicity in a concentration-dependent manner (Fig. 4F, *P* < 0.01). To show that the effect of DAS was due to Cyp2e1 inhibition, we silenced Cyp2e1 expression in primary mouse hepatocytes and HepG2 cells using siRNA (Fig. 4G and H). Our results demonstrated that the siRNA effectively moderated the induction of Cyp2e1 by PEG-ASNase treatment in both primary mouse hepatocytes (∼3-fold, Fig. 4G) and HepG2 cells (∼50%, Fig. 4H). Corresponding to the attenuated induction of Cyp2e1, we also demonstrated that the genetic inhibition of Cyp2e1 using siRNA significantly protected primary hepatocytes and HepG2 cells from PEG-ASNase-induced sensitization to lipotoxicity (Fig. 4I and J, *P* < 0.01), consistent with our data using DAS. Taken together, our data suggest the hypothesis that PEG-ASNase may sensitize hepatocytes to lipotoxicity by upregulating Cyp2e1, increasing ROS levels, and leading to cell death.

## 4 Discussion

PEG-ASNase is a critical antileukemic agent in the treatment of pediatric ALL. Despite evidence supporting its efficacy for adult ALL, the chemotherapeutic agent is not widely used in adult ALL clinical practice due to dose-limiting liver injury. The current study builds upon our prior research suggesting an association between adipocyte lipolysis induced by PEG-ASNase and its risk of liver injury^7^. Specifically, our study not only shows that PEG-ASNase-induced liver injury is reproducible in C57BL/6 mice (Supporting Information Fig. S1A–S1I), but more importantly, demonstrates that PEG-ASNase increases p-ATGL levels (Fig. 1B and C). We further demonstrated that PEG-ASNase leads to lipotoxicity in mice (Fig. 1A) and sensitizes hepatocytes to lipid-mediated toxicity (Fig. 2A–C), representing a significant novel discovery. Our RNA-seq analysis identified the upregulation of Cyp2e1 by PEG-ASNase as a potential mediator in the mechanism of sensitization to lipotoxicity (Fig. 3A–C), a novel finding further supported by our pharmacological and genetic Cyp2e1 inhibition studies (Fig. 4F–J), making this the first study to establish a connection between Cyp2e1 and PEG-ASNase-induced toxicities. Additionally, we performed translational studies using human samples to corroborate the effect of PEG-ASNase on adipocyte lipolysis and the corresponding signaling cascade in both mice and humans. While several previous clinical studies have identified an association between ASNase and the development of hypertriglyceridemia^29^, ^30^, ^31^, no association has been found between ASNase-induced hypertriglyceridemia and liver injury^31^. This indicates that ASNase can affect triglyceride disposition through different mechanisms and emphasizes the significance of our research in elucidating the mechanisms of ASNase-induced liver injury. Taken together, our comprehensive study, including *in vitro*, *ex vivo*, and translational mouse and human models, supports our hypothesis that PEG-ASNase-induced FFA mobilization from adipose tissue increases hepatic FFA accumulation and sensitizes hepatocytes to lipotoxicity.

Our RNA-seq analysis identified drug metabolism mediated by CYP450 enzymes as a dysregulated pathway involved in the mechanism of PEG-ASNase-induced sensitization to hepatocyte lipotoxicity (Fig. 3B). While Cyp2a5 showed the most significant upregulation (Fig. 3A and C), Cyp2e1 was unique in being induced by PEG-ASNase, and further exacerbated when combined with palmitic acid, as opposed to being strongly induced by palmitic acid treatment (Fig. 3C). Cyp2e1 is mainly expressed in hepatocytes^24^, and it metabolizes a variety of substrates including saturated and polyunsaturated fatty acids^27^. The enzyme uses molecular oxygen to metabolize fatty acids, leading to the production of ROS and lipid peroxidation^26^. After validating that PEG-ASNase upregulates Cyp2e1 expression in both mice and primary hepatocytes (Fig. 4A–D), subsequent experiments with primary hepatocytes showed that the Cyp2e1 upregulation is associated with increased ROS levels (Fig. 4E). Furthermore, we demonstrated that pharmacological and genetic inhibition of Cyp2e1 mitigated the sensitization to lipotoxicity induced by PEG-ASNase. The experimental design used to reach these conclusions was based on clinically relevant and feasible conditions. We assessed differential gene expression between PA treatment alone and in combination with PEG-ASNase to identify genes involved in PEG-ASNase-induced sensitization to lipotoxicity. Nevertheless, there was an approximate 50% loss in cell viability, indicating potential survivor bias despite our careful experimental design, validation experiments, and complementary measurements. Therefore, we cannot exclude the possibility that other mechanisms may sensitize hepatocytes to lipotoxicity following PEG-ASNase treatment. Considering all factors, our results support the hypothesis that PEG-ASNase mobilizes FFAs from adipocytes and sensitizes hepatocytes to their toxicity by inducing Cyp2e1 and increasing ROS levels.

Multiple clinical studies have indicated that ASNase is associated with alterations in lipid metabolism^4^^,^^32^ and the development of hepatic steatosis^33^. Both our prior preclinical research^7^ and this study demonstrate that PEG-ASNase treatment results in increased plasma FFA levels and the development of fatty liver (Fig. S1F–S1I), consistent with clinical observations. Based on our previous studies with HepG2 cells and primary hepatocytes^7^, and corroborated by the results presented here (Fig. 2A–D), we have consistently found that PEG-ASNase is non-toxic to hepatocytes at therapeutic drug levels (1 IU/mL)^19^. Therefore, we propose that the hepatotoxic effects associated with PEG-ASNase are indirectly due to the mobilization of FFA from adipose tissue. Our hypothesis that PEG-ASNase aggravates ROS-mediated hepatotoxicity is supported by our *in vitro* studies demonstrating that PEG-ASNase exacerbates PA-induced ROS levels (Fig. 4E) and sensitizes primary hepatocytes to 4-HNE-induced cytotoxicity (Fig. 2D). Nevertheless, additional research is needed to demonstrate the effect of ASNase on human hepatocytes and to determine the effect of concomitant antileukemic agents on the risk of clinical liver injury in patients receiving asparaginase.

Altogether, we hypothesize that the mechanism of PEG-ASNase-induced liver injury involves multi-organ crosstalk between the adipose tissue and the liver. In the adipose tissue, PEG-ASNase activates ATGL, resulting in FFA mobilization and leading to hepatic steatosis. In the liver, PEG-ASNase induces the expression of Cyp2e1 and sensitizes hepatocytes to lipotoxicity, though the underlying mechanisms remain to be elucidated. However, our study has several limitations. While our experiments using DAS and Cyp2e1 siRNA in primary mouse hepatocytes and HepG2 cells indicate that inhibiting Cyp2e1 attenuates PEG-ASNase sensitization to lipotoxicity, *in vivo* genetic and pharmacological Cyp2e1 inhibition studies are needed to conclusively demonstrate the role of Cyp2e1 in hepatic ROS levels and cell death induced by PEG-ASNase. Additionally, although we observed significant protection against PEG-ASNase-induced lipotoxicity corresponding to the degree of Cyp2e1 inhibition by siRNA, our results did not show complete protection. Thus, we cannot exclude the involvement of other factors in PEG-ASNase sensitization to lipotoxicity. Studies using hepatocyte-specific Cyp2e1-deficient mice would provide clearer insights into the role of Cyp2e1 in PEG-ASNase-induced lipotoxicity sensitization and identify other genes involved in this process. Furthermore, while we demonstrate that PEG-ASNase induces lipolysis and increases phosphorylated ATGL, additional studies are needed to thoroughly investigate the role of adipocyte ATGL in PEG-ASNase-induced liver injury and to elucidate the mechanism by which PEG-ASNase leads to increased phosphorylation of ATGL. While our murine model of ASNase-induced liver injury recapitulates many elements of clinical toxicity, it demonstrates a modest degree of liver injury, characterized by a ∼2-fold increase in various plasma biomarkers of liver injury, hepatic steatosis, and hepatocyte apoptosis. We anticipate that elucidating the mechanism of PEG-ASNase-induced liver injury will provide a basis for understanding how leukemia and concomitant medications used during therapy interact to lead to severe hepatotoxicity.

## 5 Conclusions

In summary, our findings support that PEG-ASNase activates adipocyte ATGL, induces adipose tissue lipolysis, and leads to hepatic lipotoxicity. We demonstrate that in the liver, PEG-ASNase induces the expression of Cyp2e1 and sensitizes hepatocytes to lipotoxicity. While further mechanistic experiments are necessary to fully establish the roles of ATGL and Cyp2e1 on PEG-ASNase-induced liver injury, our results support that inhibiting either enzyme may protect against drug-induced liver injury. Future investigations will mechanistically explore the roles of ATGL and Cyp2e1 in PEG-ASNase-induced liver injury, including how the antileukemic agent induces their expression. Additionally, subsequent research will evaluate the effects of PEG-ASNase, along with other concurrent antileukemic agents or leukemia. These future studies aim to identify pharmacological targets that can ensure the safe use of this agent in patients without compromising its antileukemic effectiveness.

## Author contributions

Yin Zhu: Writing – review & editing, Writing – original draft, Visualization, Validation, Methodology, Formal analysis, Data curation, Conceptualization. Yuyin Wang: Writing – review & editing, Data curation. Keito Hoshitsuki: Writing – review & editing, Conceptualization. Da Yang: Writing – review & editing, Validation. Lauren Kokai: Writing – review & editing, Data curation, Conceptualization. Xiaochao Ma: Writing – review & editing, Conceptualization. Wen Xie: Writing – review & editing, Conceptualization. Christian A. Fernandez: Writing – review & editing, Writing – original draft, Project administration, Data curation, Conceptualization.

## Conflicts of interest

The authors have no financial or other issues that might lead to a conflict of interest.

## Appendix A Supporting information

The following are the Supporting information to this article:

## References

[1] Fu C.H., Sakamoto K.M. (2007). PEG-asparaginase. Expert Opin Pharmacother.

[2] Dinndorf P.A., Gootenberg J., Cohen M.H., Keegan P., Pazdur R. (2007). FDA drug approval summary: pegaspargase (oncaspar) for the first-line treatment of children with acute lymphoblastic leukemia (ALL). Oncologist.

[3] Kamal N., Koh C., Samala N., Fontana R.J., Stolz A., Durazo F. (2019). Asparaginase-induced hepatotoxicity: rapid development of cholestasis and hepatic steatosis. Hepatol Int.

[4] Oettgen H.F., Stephenson P.A., Schwartz M.K., Leeper R.D., Tallai L., Tan C.C. (1970). Toxicity of *E. coli*l-asparaginase in man. Cancer.

[5] Sallan S.E. (2006). Myths and lessons from the adult/pediatric interface in acute lymphoblastic leukemia. Hematology.

[6] Liu Y., Fernandez C.A., Smith C., Yang W., Cheng C., Panetta J.C. (2017). Genome-wide study links PNPLA3 variant with elevated hepatic transaminase after acute lymphoblastic leukemia therapy. Clin Pharmacol Ther.

[7] Kumar G.V.N., Hoshitsuki K., Rathod S., Ramsey M.J., Kokai L., Kershaw E.E. (2021). Mechanistic studies of PEG-asparaginase-induced liver injury and hepatic steatosis in mice. Acta Pharm Sin B.

[8] Raju J., Bird R.P. (2006). Alleviation of hepatic steatosis accompanied by modulation of plasma and liver TNF-alpha levels by *Trigonella foenum* graecum (fenugreek) seeds in Zucker obese (*fa/fa*) rats. Int J Obes.

[9] Charni-Natan M., Goldstein I. (2020). Protocol for primary mouse hepatocyte isolation. STAR Protoc.

[10] Dou X., Li S., Wang Z., Gu D., Shen C., Yao T. (2012). Inhibition of NF-*κ*B activation by 4-hydroxynonenal contributes to liver injury in a mouse model of alcoholic liver disease. Am J Pathol.

[11] Liu K.L., Chen H.W., Wang R.Y., Lei Y.P., Sheen L.Y., Lii C.K. (2006). DATS reduces LPS-induced iNOS expression, NO production, oxidative stress, and NF-*κ*B activation in RAW 264.7 macrophages. J Agric Food Chem.

[12] Caro A.A., Adlong L.W., Crocker S.J., Gardner M.W., Luikart E.F., Gron L.U. (2012). Effect of garlic-derived organosulfur compounds on mitochondrial function and integrity in isolated mouse liver mitochondria. Toxicol Lett.

[13] Ihunnah C.A., Wada T., Philips B.J., Ravuri S.K., Gibbs R.B., Kirisci L. (2014). Estrogen sulfotransferase/SULT1E1 promotes human adipogenesis. Mol Cell Biol.

[14] Kim D., Paggi J.M., Park C., Bennett C., Salzberg S.L. (2019). Graph-based genome alignment and genotyping with HISAT2 and HISAT-genotype. Nat Biotechnol.

[15] Love M.I., Huber W., Anders S. (2014). Moderated estimation of fold change and dispersion for RNA-seq data with DESeq2. Genome Biol.

[16] Ayala A., Munoz M.F., Arguelles S. (2014). Lipid peroxidation: production, metabolism, and signaling mechanisms of malondialdehyde and 4-hydroxy-2-nonenal. Oxid Med Cell Longev.

[17] Ahmadian M., Abbott M.J., Tang T., Hudak C.S., Kim Y., Bruss M. (2011). Desnutrin/ATGL is regulated by AMPK and is required for a brown adipose phenotype. Cell Metab.

[18] Nielsen T.S., Jessen N., Jorgensen J.O., Moller N., Lund S. (2014). Dissecting adipose tissue lipolysis: molecular regulation and implications for metabolic disease. J Mol Endocrinol.

[19] Riccardi R., Holcenberg J.S., Glaubiger D.L., Wood J.H., Poplack D.G. (1981). l-Asparaginase pharmacokinetics and asparagine levels in cerebrospinal fluid of rhesus monkeys and humans. Cancer Res.

[20] Panetta J.C., Gajjar A., Hijiya N., Hak L.J., Cheng C., Liu W. (2009). Comparison of native *E. coli* and PEG asparaginase pharmacokinetics and pharmacodynamics in pediatric acute lymphoblastic leukemia. Clin Pharmacol Ther.

[21] Avramis V.I., Spence S.A. (2007). Clinical pharmacology of asparaginases in the United States: asparaginase population pharmacokinetic and pharmacodynamic (PK–PD) models (NONMEM) in adult and pediatric ALL patients. J Pediatr Hematol Oncol.

[22] Carta G., Murru E., Banni S., Manca C. (2017). Palmitic acid: physiological role, metabolism and nutritional implications. Front Physiol.

[23] Hauck A.K., Bernlohr D.A. (2016). Oxidative stress and lipotoxicity. J Lipid Res.

[24] Lu Y., Cederbaum A.I. (2008). CYP2E1 and oxidative liver injury by alcohol. Free Radic Biol Med.

[25] Lu Y., Zhuge J., Wu D., Cederbaum A.I. (2011). Ethanol induction of CYP2A5: permissive role for CYP2E1. Drug Metab Dispos.

[26] Leung T.M., Nieto N. (2013). CYP2E1 and oxidant stress in alcoholic and non-alcoholic fatty liver disease. J Hepatol.

[27] Laethem R.M., Balazy M., Falck J.R., Laethem C.L., Koop D.R. (1993). Formation of 19(*S*)-, 19(*R*)-, and 18(*R*)-hydroxyeicosatetraenoic acids by alcohol-inducible cytochrome P450 2E1. J Biol Chem.

[28] Rao P.S., Midde N.M., Miller D.D., Chauhan S., Kumar A., Kumar S. (2015). Diallyl sulfide: potential use in novel therapeutic interventions in alcohol, drugs, and disease mediated cellular toxicity by targeting cytochrome P450 2E1. Curr Drug Metab.

[29] Hoogerbrugge N., Jansen H., Hoogerbrugge P.M. (1997). Transient hyperlipidemia during treatment of ALL with l-asparaginase is related to decreased lipoprotein lipase activity. Leukemia.

[30] Finch E.R., Smith C.A., Yang W., Liu Y., Kornegay N.M., Panetta J.C. (2020). Asparaginase formulation impacts hypertriglyceridemia during therapy for acute lymphoblastic leukemia. Pediatr Blood Cancer.

[31] Bhojwani D., Darbandi R., Pei D., Ramsey L.B., Chemaitilly W., Sandlund J.T. (2014). Severe hypertriglyceridaemia during therapy for childhood acute lymphoblastic leukaemia. Eur J Cancer.

[32] Saito T., Wei Y., Wen L., Srinivasan C., Wolthers B.O., Tsai C.Y. (2021). Impact of acute lymphoblastic leukemia induction therapy: findings from metabolomics on non-fasted plasma samples from a biorepository. Metabolomics.

[33] Pratt C.B., Johnson W.W. (1971). Duration and severity of fatty metamorphosis of the liver following l-asparaginase therapy. Cancer.

