# Supplementary material for: Induction of Cyp2e1 contributes to asparaginase-induced hepatocyte sensitization to lipotoxicity
Source: Acta Pharm Sin B. 2024 Nov 7;15(2):963–72. doi: 10.1016/j.apsb.2024.11.002 (PMC11959929; doi:10.1016/j.apsb.2024.11.002)
Supplement: Multimedia component 1 [file mmc1.pdf]

Supporting Information for

Original article

Induction of Cyp2e1 contributes to asparaginase-induced hepatocyte sensitization to lipotoxicity

Yin Zhu[a], Yuyin Wang[a], Keito Hoshitsuki[a], Da Yang[a], Lauren Kokai[b], Xiaochao Ma[a], Wen Xie[a], Christian A. Fernandez[a]

[a]*Center for Pharmacogenetics and Department of Pharmaceutical Sciences, University of Pittsburgh, Pittsburgh, PA 15261, USA*

[b]*Department of Plastic Surgery, University of Pittsburgh and the McGowan Institute for Regenerative Medicine, Pittsburgh, PA 15261, USA*

Received 15 May 2024; received in revised form 13 August 2024; accepted 26 September 2024

*Corresponding author.

E-mail address: chf63@pitt.edu (Christian A. Fernandez).

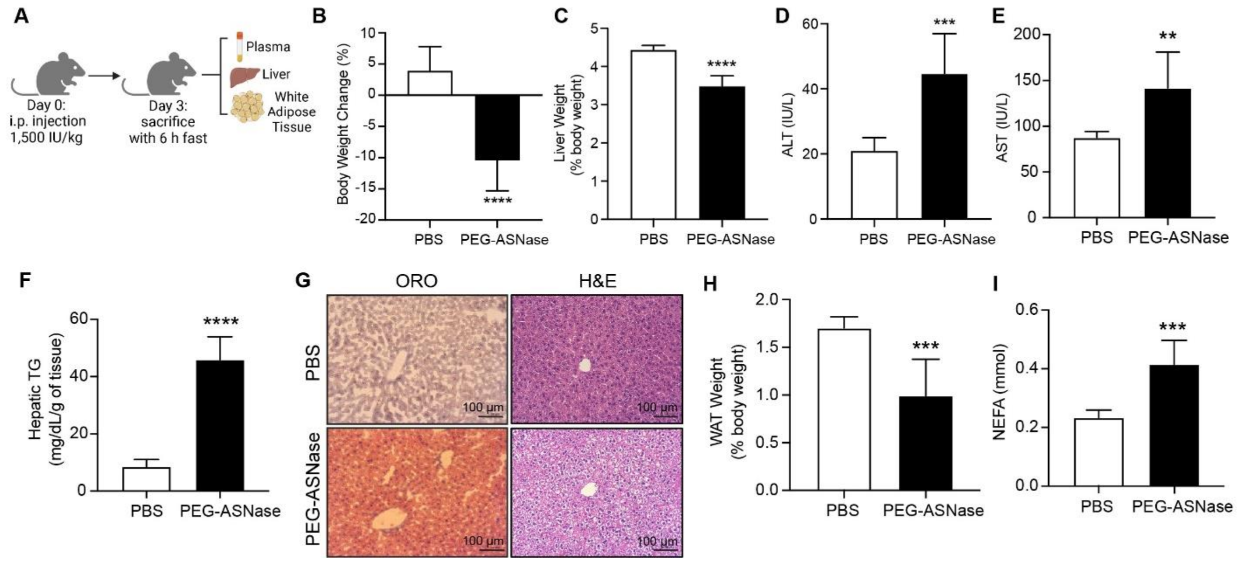

Figure S1 Mice receiving PEG-ASNase develop liver injury. (A) Schematic of PEG-ASNase-induced liver injury mouse model. (B) PEG-ASNase treatment (1500 IU/kg, IP) resulted in an 11.46% decrease in body weight compared to PBS controls ($P < 1\times10^{-4}$), and (C) an 11% reduction in normalized liver weight compared to controls ($P < 1\times10^{-4}$). Elevated levels of plasma (D) ALT, (E) AST, and (F) hepatic triglycerides (TG) were detected in PEG-ASNase-treated mice compared to control mice. (G) Oil Red O (ORO) and H&E staining of liver sections further indicated the development of fatty liver in PEG-ASNase-treated mice compared to PBS controls. (H) PEG-ASNase-treated mice showed a loss of white adipose tissue (WAT) mass and (I) elevated plasma non-esterified fatty acids (NEFA), consistent with adipose tissue free fatty acid mobilization contributing to hepatic steatosis and liver injury. *, **, ***, **** indicate $P <$ 0.05, 0.01, 0.001 and 1×10^{-4} respectively.

Table S1 Primer sequences.

Name	Forward (5′–3′)	Reverse (5′–3′)
Actb (beta-actin)	GATTACTGCTCTGGCTCCTAG	GACTCATCGTACTCCTGCTTG
Cyp2e1 (mouse)	CAGGACCTTTCCCAATTCCT	TGACTTTTCTGTGGCTTCCA
CYP2E1 (human)	ACGGTATCACCGTGACTGTGG	GCATCTCTTGCCTATCCTTGA

Table S2 Detailed information of antibodies used in Western blot.

Name	Cat. No.	Company
α-Actinin	3134	Cell Signaling Technology
β-Actin	3700	Cell Signaling Technology
p-ATGL	ab135093	Abcam
ATGL	2138	Cell Signaling Technology
CYP2E1	ab28146	Abcam
p-HSL	PA5-64494	Invitrogen
HSL	4107	Cell Signaling Technology

Table S3 Donor Human Patient Information.

Patient ID	Sex	BMI	Race	Age	Preparation process
1	Female	33.47	African American	31	Minced whole fat
2	Female	27.91	White	35	Minced whole fat
3	Female	30.36	White	42	Minced whole fat
4	Female	22.7	White	57	Liposuction fat
5	Female	29.17	White	22	Liposuction fat
6	Male	27.27	White	28	Minced whole fat
7	Female	33.2	White	34	Liposuction fat

Table S4 PNEA_PA *vs* PA dysregulated genes.

See Excel file.
